# Supplementary material for: The last-born daughter cell contributes to division orientation of Drosophila larval neuroblasts
Source: Nat Commun. 2018 Sep 14;9:3745. doi: 10.1038/s41467-018-06276-0 (PMC6138640; doi:10.1038/s41467-018-06276-0)
Supplement: Supplementary file 1 — Supplementary Information [file 41467_2018_6276_MOESM1_ESM.pdf]

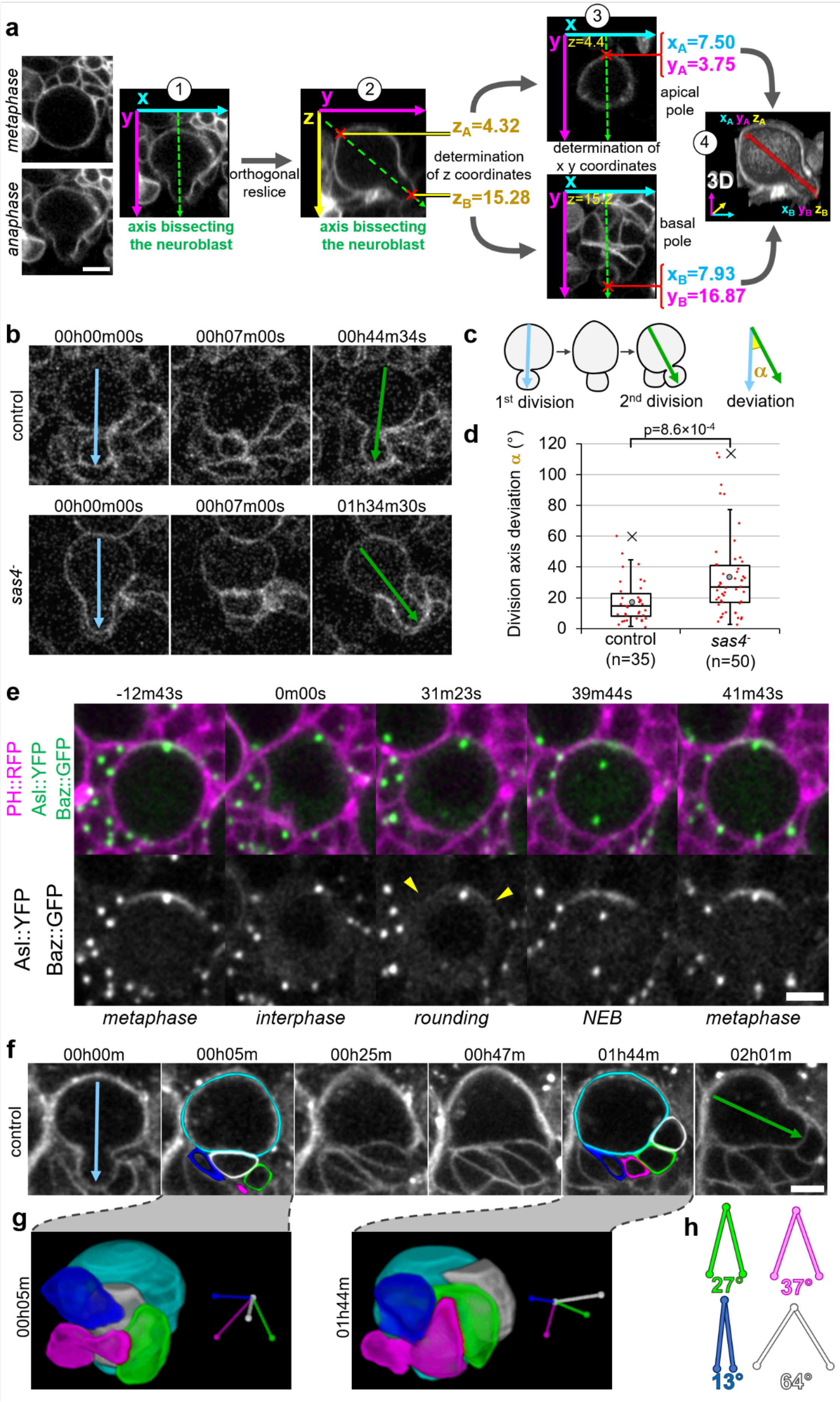

a
metaphase
anaphase
x
y
1
axis bissecting the neuroblast
orthogonal reslice
y
z
2
zA=4.32
zB=15.28
determination of z coordinates
axis bissecting the neuroblast
3
z=4.4
xA=7.50
yA=3.75
apical pole
determination of x y coordinates
z=15.2
basal pole
xB=7.93
yB=16.87
4
3D
xA yA zA
xB yB zB
b
00h00m00s
00h07m00s
00h44m34s
control
00h00m00s
00h07m00s
01h34m30s
sas4-
c
1st division
2nd division
α
deviation
d
Division axis deviation α (°)
p=8.6×10-4
120
100
80
60
40
20
0
control (n=35)
sas4- (n=50)
e
-12m43s
0m00s
31m23s
39m44s
41m43s
PH::RFP Asl::YFP Baz::GFP
Asl::YFP Baz::GFP
metaphase
interphase
rounding
NEB
metaphase
f
00h00m
00h05m
00h25m
00h47m
01h44m
02h01m
control
g
00h05m
01h44m
h
27°
37°
13°
64°

Supplementary Fig. 1: 3D division angle measurements in larval neuroblasts.

a Methodology for 3D division axis measurement. 1) At telophase, a manually determined axis bisecting the neuroblast (green arrow) is used to orthogonally slice a 3D stack covering its entire volume. 2) This orthogonal slice is used to determine the z coordinates of the apical and basal poles (red crosses). 3) These z coordinates are used on the corresponding slices of the 3D stack to determine the x and y coordinates of the basal and apical poles (red crosses). 4) 3D reconstruction of the neuroblast volume and the division axis measured with this method (red). Angles between successive division axis are calculated from these 3D coordinates (see Material and Methods). **b** Two successive divisions of control and *sas4* mutant neuroblasts expressing *worniu*-GAL4-driven PH::GFP. **c** Schematic of the angles α measured in the movies shown in the previous panels. **d** Distribution of the angles α described in the previous panels in control (18±14°, n=35, data from 10 independent experiments, *also shown in **Figure 2c***) and *sas4* mutant neuroblasts (33±26°, n=50, data from 6 independent experiments, *also shown in **Figure 3c***). **e** High time resolution movie (Dt: 23.84s) of a neuroblast expressing PH::RFP (magenta), Asl::YFP and Baz::GFP (green in merge). 0m00s corresponds to the beginning of interphase, when the neuroblast adopted a stable shape. A faint apical Baz crescent (delimited by arrowheads) is detected as soon as neuroblasts starts rounding up. **f** Manual segmentation of the neuroblast/progeny cluster shown in **Figure 1a** at the beginning of interphase (5 minutes after the first division) and the beginning of the second division, detected by mitotic rounding (1h44m after the first division). Cyan: neuroblast. White: last-born GMC. Magenta, green, blue: other neuroblast-neighbouring cells. **g** 3D reconstruction of the manual segmentation shown in the previous panel (left) and 3D vectors defined by the coordinates of the barycenter of the neuroblast (where all vectors converge) and other neighbouring cells (right). **h** Angles between the neuroblast-other cell vectors at the two time points described in the previous panel. The GMC (white) shows a higher angle than other cells, consistent with its apparent relocalisation. Scale bar in all panels: 5 µm.

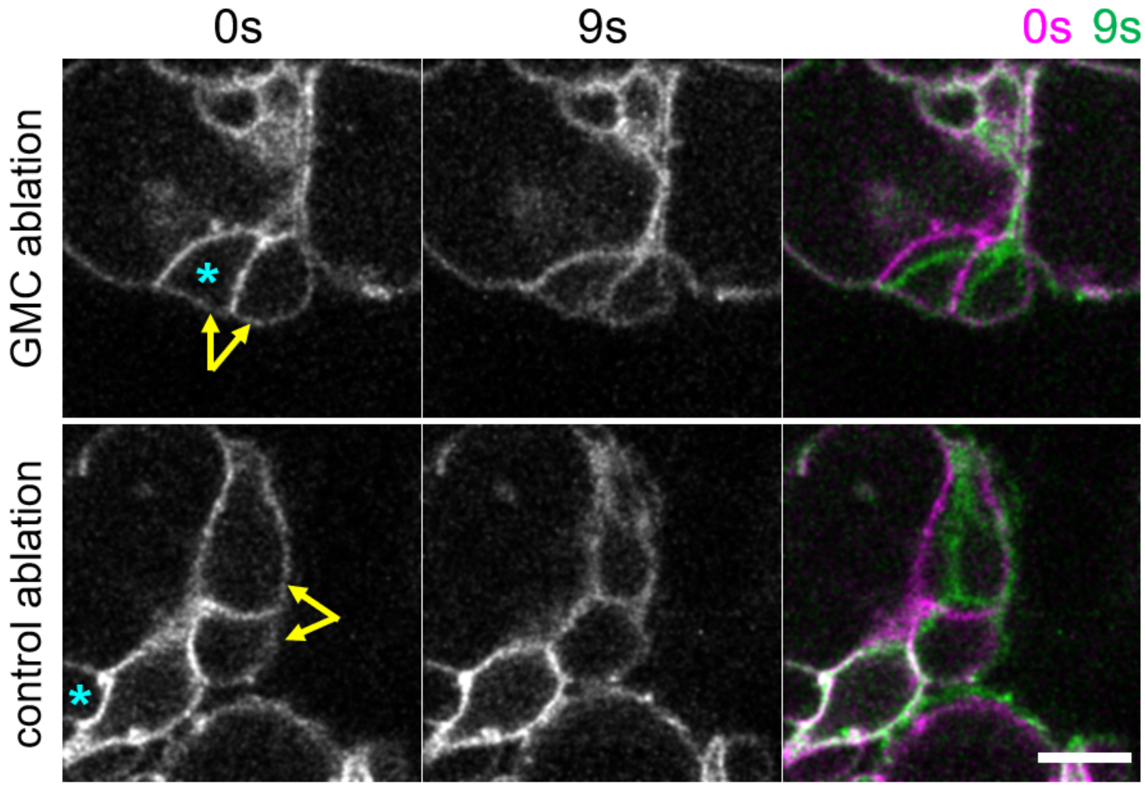

Supplementary Fig. 2: Laser ablation of neighboring cells causes neuroblast deformation. Deformation of neuroblasts expressing *worniu*-GAL4-driven PH::GFP following laser ablation, which occurs approximately 1.5 seconds after the first time point following ablation (0s). For efficient and reproducible ablations, we always targeted the interface between two cells (arrows) of the neuroblast/progeny cluster: control ablations targeted two neuroblast-neighboring cells away from the GMC, and GMC ablations targeted the GMC and another neuroblast-neighboring cell. The third panel is a composite picture of the first two panels, illustrating the immediate neuroblast deformation toward the targeted cells. Blue asterisk: last-born GMC. Yellow arrows point at relevant cells. Scale bar: 5 µm.

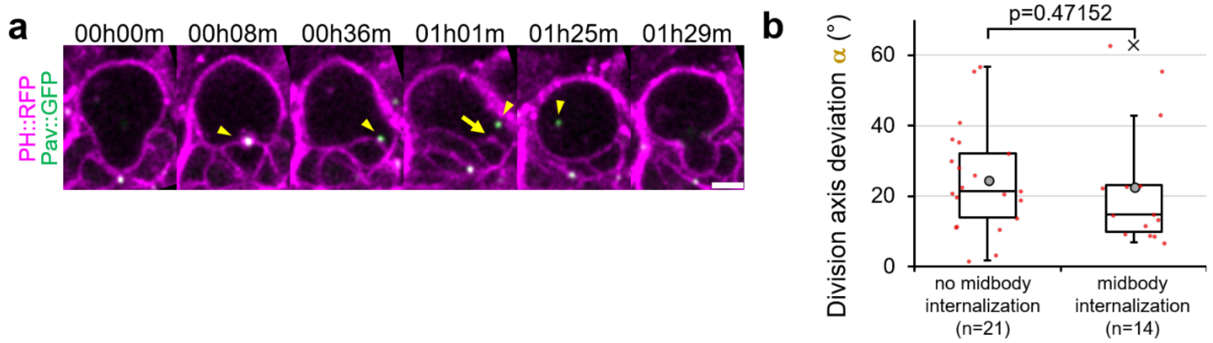

Supplementary Fig. 3: Midbody internalization does not affect neuroblast division axis maintenance.

a Two successive divisions of a neuroblast expressing the membrane marker PH::RFP (magenta) and the midbody marker Pav::GFP (green). The midbody formed during the first division (arrowhead) detaches from the neuroblast/GMC interface before the second division. Plasma membrane extensions (arrow) are still present after midbody internalization. **b** Division axis deviation when the midbody is kept at the interface until the next division (25±15°, n=21) and when the midbody is internalized before the next division (23±17, n=14). Data from one experiment. Scale bar: 5 µm.

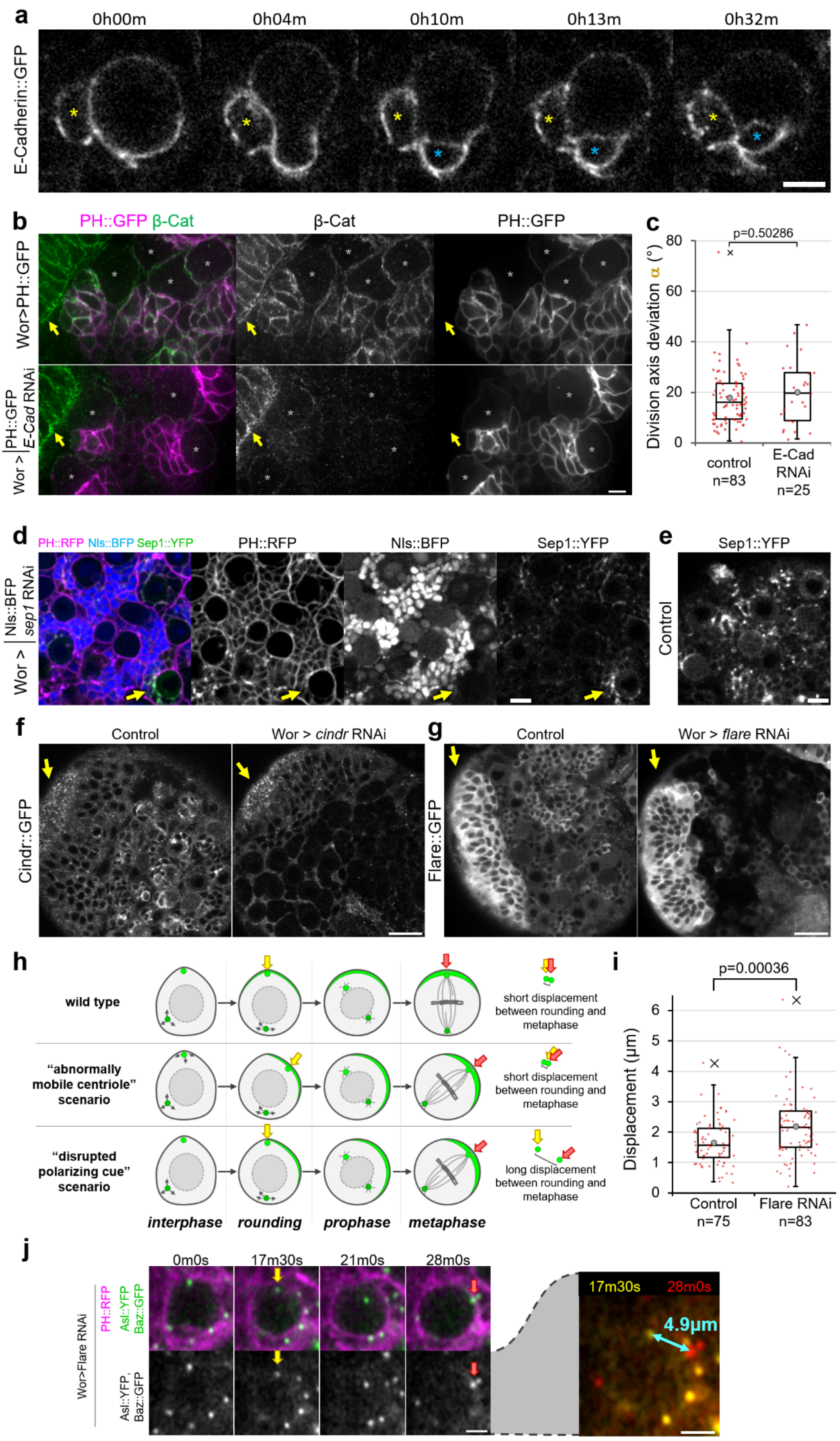

a
0h00m
0h04m
0h10m
0h13m
0h32m
E-Cadherin::GFP
b
PH::GFP β-Cat
β-Cat
PH::GFP
Wor>PH::GFP
Wor > PH::GFP E-Cad RNAi
c
p=0.50286
Division axis deviation α (°)
control n=83
E-Cad RNAi n=25
d
PH::RFP Nls::BFP Sep1::YFP
PH::RFP
Nls::BFP
Sep1::YFP
Wor > Nls::BFP sep1 RNAi
e
Sep1::YFP
Control
f
Control
Wor > cindr RNAi
Cindr::GFP
g
Control
Wor > flare RNAi
Flare::GFP
h
wild type
short displacement between rounding and metaphase
"abnormally mobile centriole" scenario
short displacement between rounding and metaphase
"disrupted polarizing cue" scenario
long displacement between rounding and metaphase
interphase
rounding
prophase
metaphase
i
p=0.00036
Displacement (µm)
Control n=75
Flare RNAi n=83
j
0m0s
17m30s
21m0s
28m0s
Wor>Flare RNAi
PH::RFP Asl::YFP Baz::GFP
Asl::YFP, Baz::GFP
17m30s
28m0s
4.9µm

Supplementary Fig. 4: RNAi-mediated depletion of components of the neuroblast/ GMC interface.

a Neuroblast expressing E-Cadherin::GFP. Yellow asterisk: last-born GMC. Blue asterisk: older GMC. Scale bar: 5µm. **b** Neuroblasts stained for β-Cat and expressing *worniu*-Gal4-driven PH::GFP and, for the bottom panel, *worniu*-Gal4-driven *E-Cadherin* RNAi. Arrows point to a neuroepithelial region of the brain, expressing β-Catenin::YFP but not *worniu*-GAL4. Scale bar: 5µm. **c** Deviation of the division angle in control (18±11°, n=83, also shown in **Figure 5a**) and E-Cad-depleted neuroblasts (20±13°, n=25). **d** Neuroblasts expressing PH::RFP, Sep1::YFP, and *worniu*-Gal4-driven Nls::BFP and *sep1* RNAi1. The arrow points to a neuroblast expressing no detectable amount of the GAL4 reporter Nls::BFP and displaying higher amounts of Sep1::YFP. Scale bar: 10 µm. **e** Control neuroblasts expressing Sep1::YFP. Scale bar: 10 µm. **f** Control and *cindr* RNAi-expressing neuroblasts expressing Cindr::GFP. The arrows point to a neuroepithelial region not expressing Wor-GAL4. Scale bar: 20 µm. **g** Control and *flare* RNAi-expressing neuroblasts expressing Flare::GFP. The arrows point to a neuroepithelial region not expressing *worniu*-GAL4. Scale bar: 20 µm. **h** Two scenarios could explain the mispositioning of Baz crescents in interface components-depleted neuroblasts: an abnormally mobile centriole directing polarization at another place, or the polarizing effect of the NB/GMC interface is disrupted. Measuring the displacement of the apical centriole from polarization (yellow arrow, as soon as neuroblasts start rounding up) to metaphase (red arrow, when the centriole always relocalizes to the center of the apical crescents) allows to discriminate between these possibilities. Centriole position in prophase was not considered, as centrioles transiently detach from the cortex at that phase. **i** Displacement of the centriole explained in G), in control (1.7±0.7µm n=75) and *flare* RNAi-expressing neuroblasts (2.2±1.0µm, n=83). The significantly higher displacement in Flare-depleted neuroblasts supports the "disrupted polarizing cue" scenario. Data from 3 independent experiments. **j E**xample of a long displacement of the apical centriole from initiation of the rounding to (yellow arrow) to metaphase (red arrow) in a neuroblast expressing *flare* RNAi. Right panel: composite picture of these two timepoints. Scale bars: 5 µm.

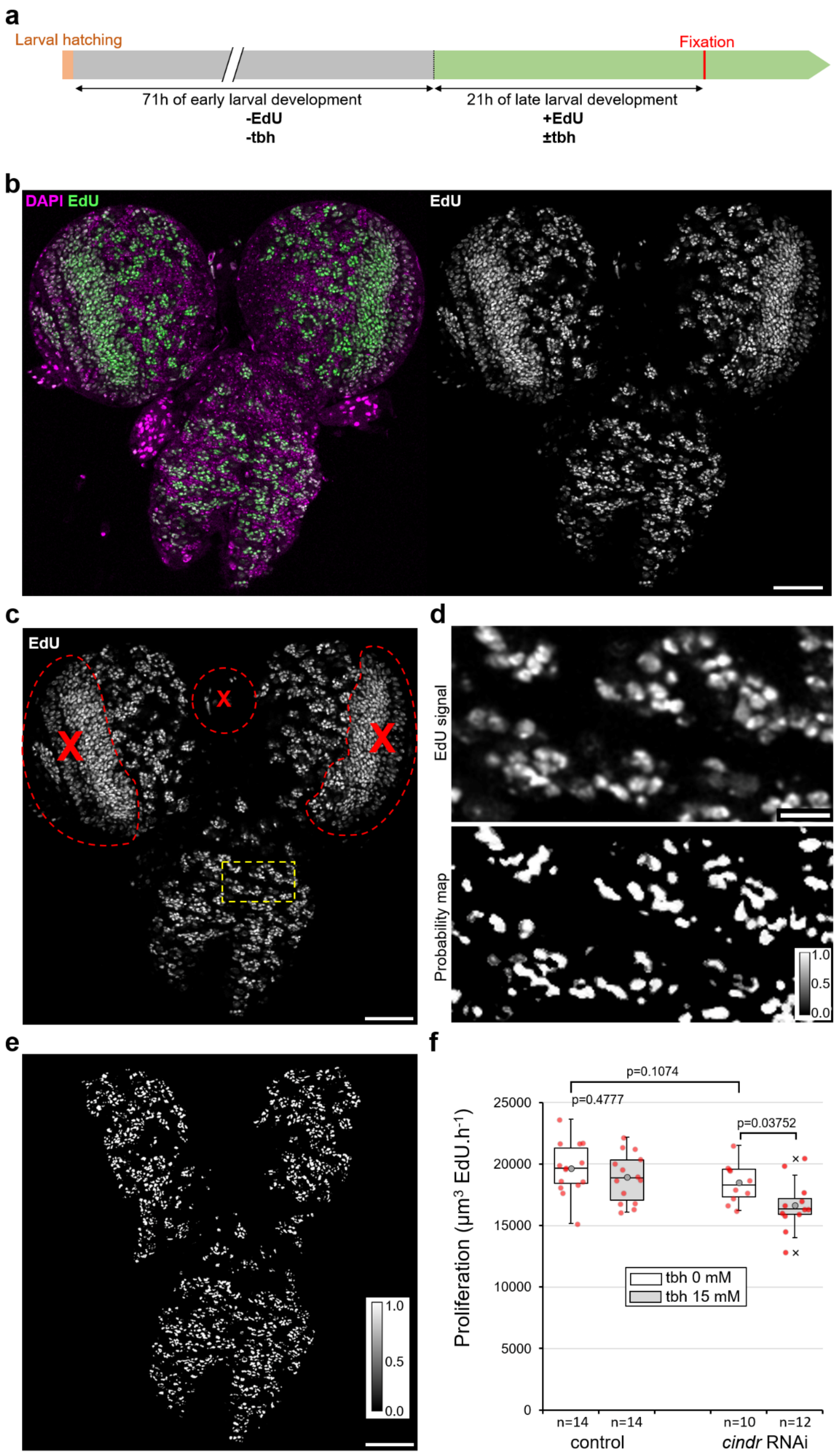
a
Larval hatching
Fixation
71h of early larval development
-EdU
-tbh
21h of late larval development
+EdU
±tbh
b
DAPI EdU
EdU
c
EdU
d
EdU signal
Probability map
1.0
0.5
0.0
e
1.0
0.5
0.0
f
Proliferation (µm³ EdU.h⁻¹)
p=0.1074
p=0.4777
p=0.03752
25000
20000
15000
10000
5000
0
tbh 0 mM
tbh 15 mM
n=14
n=14
n=10
n=12
control
cindr RNAi

Supplementary Fig.5: Neuroblasts with defective division axis maintenance are more sensitive to oxidative stress

a Proliferation assay. Larvae are fed on standard medium until 71h after larval hatching, then fed for 21 hours on standard medium containing EdU, with or without 15mM of the oxidant tbh, after which their brains are fixed and the incorporated EdU is revealed. **b** Larval brain following 21h of feeding on standard medium containing EdU (green), labelling neuroblasts and their progeny born during this period. Magenta: DAPI staining. Scale bar: 50 µm. **c** The central brain and ventral nerve chord were segmented post-acquisition by manually deleting the signal from the optic lobes (larger regions circled by a red line) and any other tissue possibly remaining from the dissection (smaller region circled by a red line). 3D regions such as the one circled in yellow were used to train the Ilastik software for segmentation (see next panel). Scale bar: 50 µm. **d** The Pixel Classification workflow of Ilastik was trained on small regions to accurately detect and segment the EdU signal, by generating a signal probability map. Scale bar: 10 µm. **e** Resulting signal probability map of the entire brain, after deletion of the unwanted regions highlighted in panel C). The map was ultimately segmented into a binary image by applying a threshold of 0.5 (not shown), and used to measure the volume of incorporated EdU. Scale bar: 50 µm. **f** Volume of incorporated EdU in control and cindr RNAi brains, in the presence or absence of the oxidant tbh. Data from 6 independent experiments.

Supplementary Table 1. Genotypes

**Figure**	**Genotype**
1a, S1f, 4a, S3a	+ ; Ubi-*PH::RFP* / CyO ; Ubi-*pav::GFP* / TM6 Tb, Hu
S1a, 2a-d, 3c GMC ablation, S2, 5c GMC ablation	+ ; Wor-*GAL4*, UAS-*PH::GFP* / + ; + / MKRS
S1b control	+ ; Wor-*GAL4*, UAS-*PH::GFP* / + ; FRT[82B], *sas4*^{s2214} / TM6 Tb, Hu
S1b *sas4*$^{-}$,3c *sas*$^{-}$	+ ; Wor-*GAL4*, UAS-*PH::GFP* / + ; FRT[82B], *sas4*^{s2214}
3a	Ubi-*asl::YFP*, *baz::GFP*^{CC01941} ; Wor-*GAL4*, Ubi-*PH::RFP* ; UAS-*His3.3::mIFP-T2A-HO1*
4b	*rap1::GFP*/Sm6a
4c	Ubi-*PH::RFP* ; Sep2-*sep2::GFP*
4d	Sqh-*utr::GFP* and *cno::YFP*^{CPTI000590} and *flr::GFP*^{CA07499} and *cindr::GFP*^{CA06686}
5a control, S1e, S4c control, S4i control	Ubi-*asl::YFP*, *baz::GFP*^{CC01941} / + ; Wor-*GAL4*, Ubi-*PH::RFP* / + ; UAS-*nls::TagBFP* / +
5a Sep1 RNAi	Ubi-*asl::YFP*, *baz::GFP*^{CC01941} / + ; Wor-*GAL4*, Ubi-*PH::RFP* / + ; UAS-*nls::TagBFP* / UAS-*sep1 RNAi*$^{TRiP.JF02789}$
5a Sep2 RNAi	Ubi-*asl::YFP*, *baz::GFP*^{CC01941} / + ; Wor-*GAL4*, Ubi-*PH::RFP* / + ; UAS-*nls::TagBFP* / UAS-*sep2 RNAI*$^{TRiP.JF02838}$
5a Cindr RNAi, 5b	Ubi-*asl::YFP*, *baz::GFP*^{CC01941} / + ; Wor-*GAL4*, Ubi-*PH::RFP* / UAS-*cindr RNAI*$^{TRiP.HMS01892}$; UAS-*nls::TagBFP* / +
5a Dock RNAi	Ubi-*asl::YFP*, *baz::GFP*^{CC01941} / + ; Wor-*GAL4*, Ubi-*PH::RFP* / + ; UAS-*nls::TagBFP* / UAS-*dock RNAI*$^{TRiP.GL01519}$
5a Rst RNAi	Ubi-*asl::YFP*, *baz::GFP*^{CC01941} / + ; Wor-*GAL4*, Ubi-*PH::RFP* / + ; UAS-*nls::TagBFP* / UAS-*rst RNAi*$^{\{GD14475\}v27223}$
5a Flare RNAi, S4i Flare RNAi	Ubi-*asl::YFP*, *baz::GFP*^{CC01941} / + ; Wor-*GAL4*, Ubi-*PH::RFP* / UAS-*flr RNAi*^{KK100083} ; UAS-*nls::TagBFP* / +
5c Sep1 RNAi	+ ; Wor-*GAL4*, UAS-*PH::GFP* / + ; UAS-*sep1 RNAi*$^{TRiP.JF02789}$ / MKRS
6b-c, S5f control	+ ; Wor-GAL4, UAS-*PH::GFP* / + ; + / MKRS
6b-c, S5f Cindr RNAi	+ ; Wor-GAL4, UAS-*PH::GFP* / UAS-*cindr RNAI*$^{TRiP.HMS01892}$; + / MKRS
S4a	*shg::GFP*
S4b control	+ ; Wor-*GAL4*, UAS-*PH::GFP* / + ; + / MKRS
S4b-c E-Cad RNAi	+ ; Wor-*GAL4*, UAS-*PH::GFP* / + ; UAS-*shg RNAi*$^{TRiP.HMS0069}$ / MKRS
S4d	*sep1::YFP*^{CPTI003593} / + ; Wor-*GAL4*, Ubi-*PH::RFP* / + ; UAS-*nls::TagBFP* / UAS-*sep1 RNAi*$^{TRiP.JF02789}$
S4e	*sep1::YFP*^{CPTI003593} / +
S4f Cindr RNAi	+ ; Wor-*GAL4*, Ubi-*PH::RFP* / UAS-*cindr RNAI*$^{TRiP.HMS01892}$; *cindr::GFP*^{CA06686} / +
S4f control	*cindr::GFP*^{CA06686} / +
S4g-h Flare RNAi	+ ; Wor-*GAL4*, Ubi-*PH::RFP* / + ; *flr::GFP*^{CA07499} / UAS-*flr RNAi*^{KK100083}
S4g-h control	*flr::GFP*^{CA07499} / +

Supplementary Table 2. Stocks origins

Ubi-*PH*$^{PLC\delta1}$*::RFP* / CyO	Claret et al., 2014 [1]
Ubi-*pav::GFP* / TM6, Tb, Hu	Minesttrini et al., 2002 [2]
UAS-*PH::GFP* (II)	BDSC 39693
FRT[82B], *sas4*^{s2214} / TM6, Tb, Hu	Basto et al., 2006 [3]
Ubi-*asl::YFP*	Rebollo et al., 2007 [4]
baz::GFP^{CC01941}	BDSC 51572
UAS-*His3.3::mIFP-T2A-HO1*	BDSC 64184
Rap1-*rap1::GFP*/Sm6a	Knox & Brown, 2002 [5]
Sep2-*sep2::GFP*	Silverman-Gavrila et al., 2008 [6]
Sqh-*utr::GFP*	Rauzi et al., 2010 [7]
cno::YFP^{CPTI000590}	DGRC 115111
flr::GFP^{CA07499}	BDSC 50824
cindr::GFP^{CA06686}	BDSC 50802
sep1::YFP^{CPTI003593}	DGRC 115423
UAS-*nls::TagBFP* / TM6, Tb, Hu	BDSC 55094
UAS-*sep1 RNAi*$^{TRiP.JF02789}$	BDSC 27709
UAS-*sep2 RNAI*$^{TRiP.JF02838}$	BDSC 28004
UAS-*cindr RNAI*$^{TRiP.HMS01892}$	BDSC 38976
UAS-*dock RNAI*$^{TRiP.GL01519}$	BDSC 43176
UAS-*rst RNAi*$^{\{GD14475\}v27223}$	VDRC 27223
UAS-*flr RNAi*^{KK100083}	VDRC 108442
shg::GFP	BDSC 60584
UAS-*shg RNAi*$^{TRiP.HMS0069}$	BDSC 32904

Supplementary References

1. Claret, S., Jouette, J., Benoit, B., Legent, K. & Guichet, A. PI(4,5)P2 produced by the PI4P5K SKTL controls apical size by tethering PAR-3 in Drosophila epithelial cells. *Curr Biol* **24,** 1071–1079 (2014).
2. Minestrini, G., Máthé, E. & Glover, D. M. Domains of the Pavarotti kinesin-like protein that direct its subcellular distribution: effects of mislocalisation on the tubulin and actin cytoskeleton during Drosophila oogenesis. *Journal of Cell Science* **115,** 725–736 (2002).
3. Basto, R. *et al.* Flies without centrioles. *Cell* **125,** 1375–1386 (2006).
4. Rebollo, E. *et al.* Functionally Unequal Centrosomes Drive Spindle Orientation in Asymmetrically Dividing Drosophila Neural Stem Cells. *Developmental Cell* **12,** 467–474 (2007).
5. Knox, A. L. & Brown, N. H. Rap1 GTPase regulation of adherens junction positioning and cell adhesion. *Science* **295,** 1285–1288 (2002).
6. Silverman-Gavrila, R. V., Hales, K. G., Hales, K. G. & Wilde, A. Anillin-mediated targeting of peanut to pseudocleavage furrows is regulated by the GTPase Ran. *Mol Biol Cell* **19,** 3735–3744 (2008).
7. Rauzi, M., Lenne, P.-F. & Lecuit, T. Planar polarized actomyosin contractile flows control epithelial junction remodelling. *Nature* **468,** 1110–1114 (2010).
