# Supplementary material for: The last-born daughter cell contributes to division orientation of Drosophila larval neuroblasts
Source: Nat Commun. 2018 Sep 14;9:3745. doi: 10.1038/s41467-018-06276-0 (PMC6138640; doi:10.1038/s41467-018-06276-0)
Supplement: Supplementary file 3 — Description of Additional Supplementary Files [file 41467_2018_6276_MOESM3_ESM.pdf]

Description of Additional Supplementary Files

File Name: Supplementary Movie 1
Description: Three successive divisions of a neuroblast expressing Worniu-GAL4-driven PH::GFP, shown in **Supplementary Figure 1A**. Middle panel: orthogonal slice along the y axis of the left panel. Right panel: 3D reconstruction of the neuroblast volume, sliced in half through its division axis. Time in hh:mm:ss, scale bar: 5 μm.

File Name: Supplementary Movie 2
Description: Two successive divisions of a neuroblast expressing PH::RFP (magenta), the centriole marker Asl::YFP and the apical polarity marker Baz::GFP (green), shown in **Supplementary Figure 1C**. Baz::GFP starts accumulating apically as the neuroblast starts rounding up, around 00:30:00. Arrow: apical centriole. Arrowheads: limits of the apical Baz crescent. Time in hh:mm:ss, scale bar: 5 μm.

File Name: Supplementary Movie 3
Description: Immediate deformation of a neuroblast expressing Worniu-GAL4-driven PH::GFP, following the ablation of its last daughter cell (occurring at 0s, red lightning symbol). Scale bar: 5 μm

File Name: Supplementary Movie 4
Description: Two successive divisions of neuroblasts expressing Worniu-GAL4-driven PH::GFP, following control ablation (red lightning symbol). Arrows: division axis. Time in hh:mm:ss, scale bar: 5 μm.

File Name: Supplementary Movie 5
Description: Two successive divisions of neuroblasts expressing Worniu-GAL4-driven PH::GFP, following last-born GMC ablation (red lightning symbol). Arrows: division axis. Time in hh:mm:ss, scale bar: 5 μm.

File Name: Supplementary Movie 6
Description: Two successive divisions of neuroblasts expressing Worniu-GAL4-driven PH::GFP, following control ablation (red lightning symbol) and ending in a high misalignment of the second division axis. Arrows: division axis. Time in hh:mm:ss, scale bar: 5 μm.

File Name: Supplementary Movie 7
Description: Two successive divisions of neuroblasts expressing Worniu-GAL4-driven PH::GFP, following last-born GMC ablation (red lightning symbol) and ending in a high misalignment of the second division axis. Arrows: division axis. Time in hh:mm:ss, scale bar: 5 μm.

File Name: Supplementary Movie 8
Description: Two successive divisions of a neuroblast expressing Worniu-GAL4-driven PH::GFP, ending after the formation of plasma membrane extensions at the neuroblast/last-born GMC interface, followed by a high-resolution series of confocal slices through the neuroblast volume, followed by a 3D reconstruction of this volume. Time in hh:mm:ss, scale bar: 5 μm.

File Name: Supplementary Movie 9
Description: Neuroblast expressing Cindr::GFP at endogenous levels. Cindr::GFP localizes to the neuroblast/GMC interface, to the plasma membrane extensions originating from it and to centrosomes. Time in hh:mm:ss, scale bar: 5 μm.

File Name: Supplementary Movie 10
Description: Two successive divisions of a neuroblast expressing PH::RFP (magenta), the centriole marker Asl::YFP, the apical polarity marker Baz::GFP (green), and Worniu-GAL4-driven *cindr* RNAi, shown in **Figure 5B**. Following the first division, the apical centrosome maintains its position throughout interphase, a Baz crescent forms at an abnormal location during cellular rounding, the spindle properly aligns with this crescent and the neuroblast divides with a 53° deviation from the first division axis. Arrow: apical centriole. Arrowheads: limits of the apical Baz crescent. Time in hh:mm:ss, scale bar: 5 μm.
